# Supplementary material for: Knock-Down of HDAC2 in Human Induced Pluripotent Stem Cell Derived Neurons Improves Neuronal Mitochondrial Dynamics, Neuronal Maturation and Reduces Amyloid Beta Peptides
Source: Int J Mol Sci. 2021 Mar 3;22(5):2526. doi: 10.3390/ijms22052526 (PMC7959288; doi:10.3390/ijms22052526)
Supplement: Supplementary file 1 [file ijms-22-02526-s001.pdf]

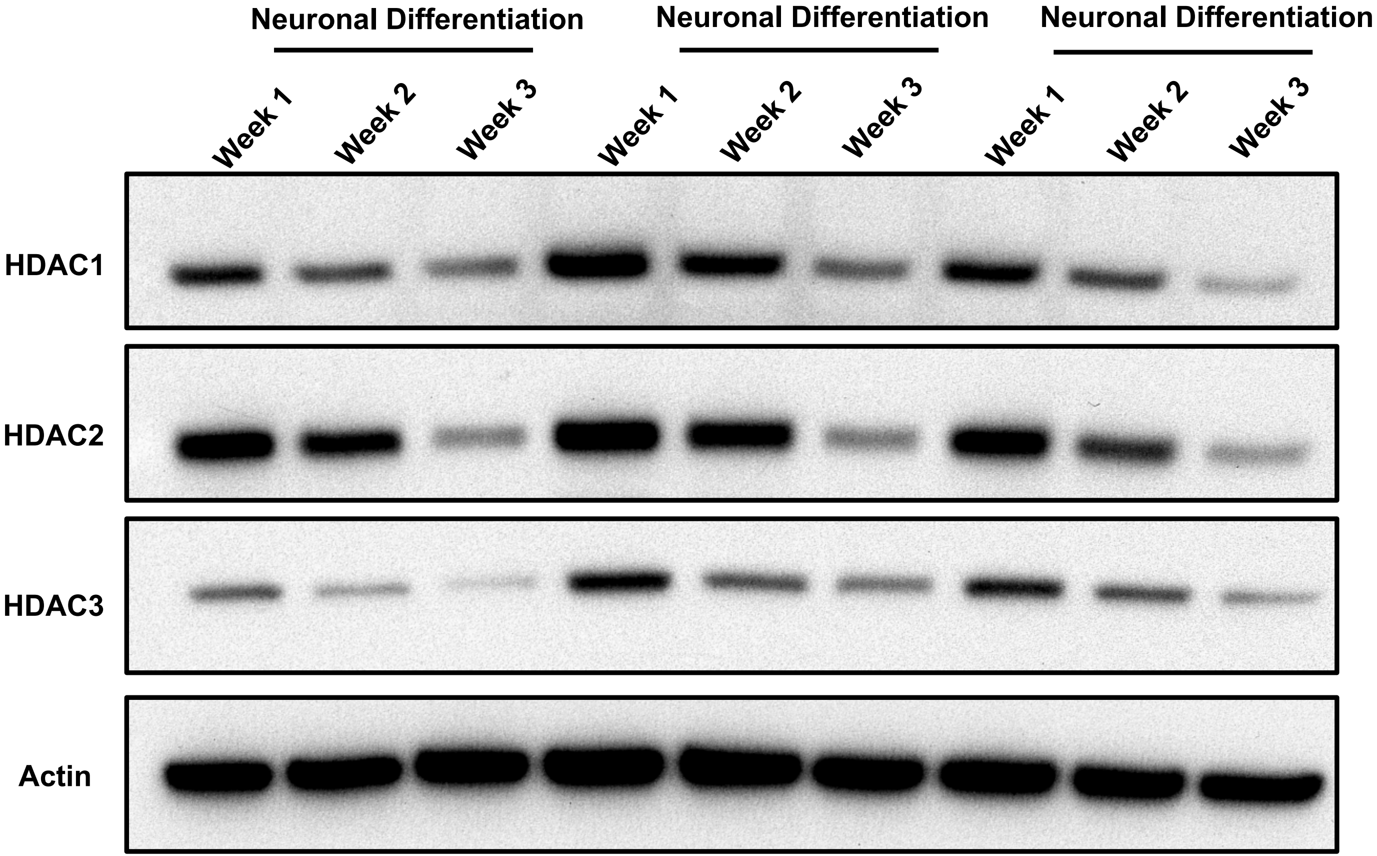

Supplemental Figure S1. Western blot of protein lysates from differentiatiating hiPSC-Ns shows that all Class I HDACs (HDAC1, HDAC2, HDAC3) decrease with neuronal [illegible]

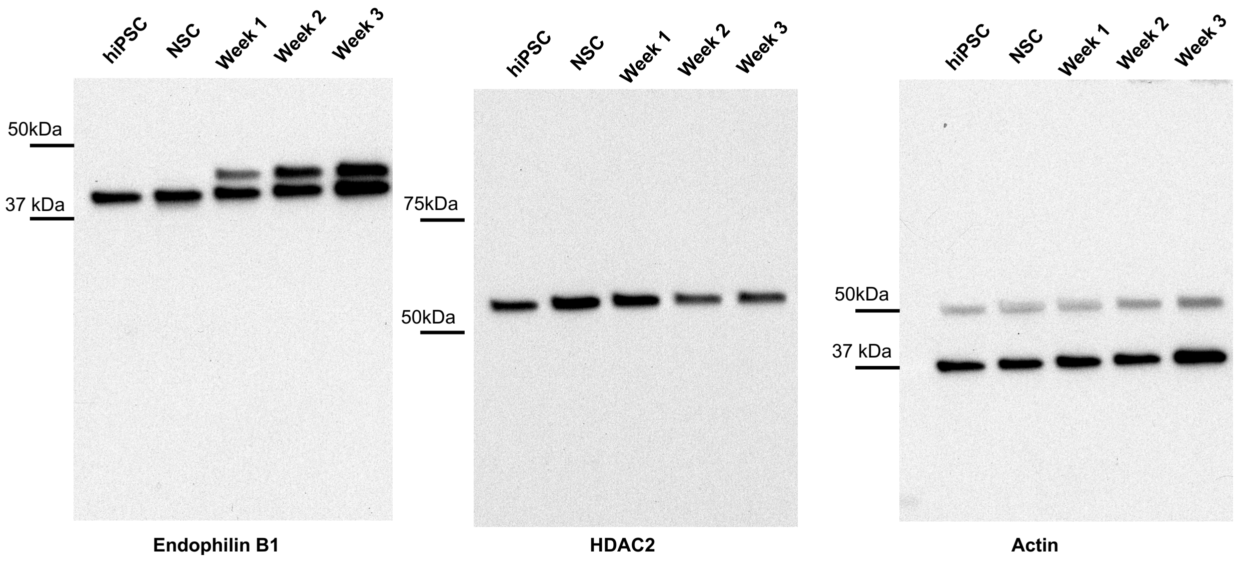

Western Blot: Fig 1

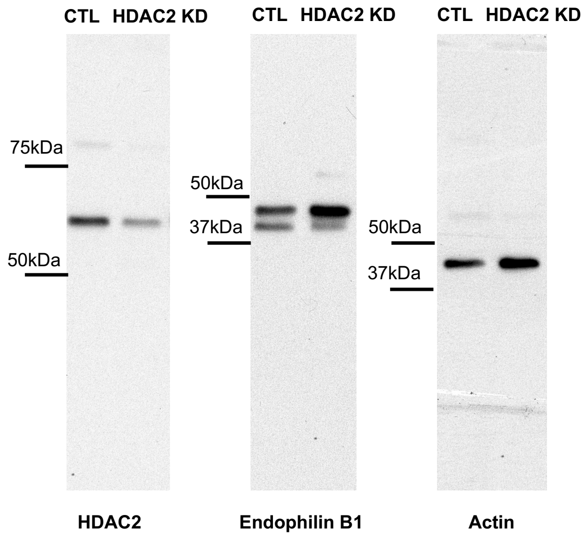

Western Blot: Fig 3

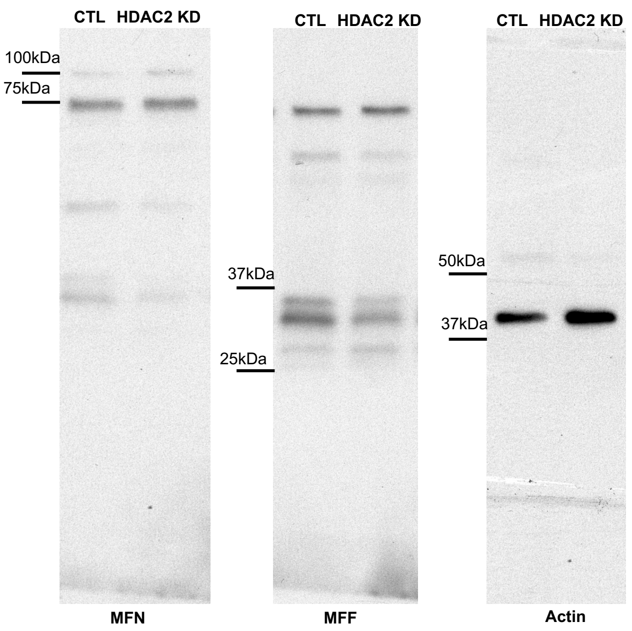

Western Blot: Fig 4

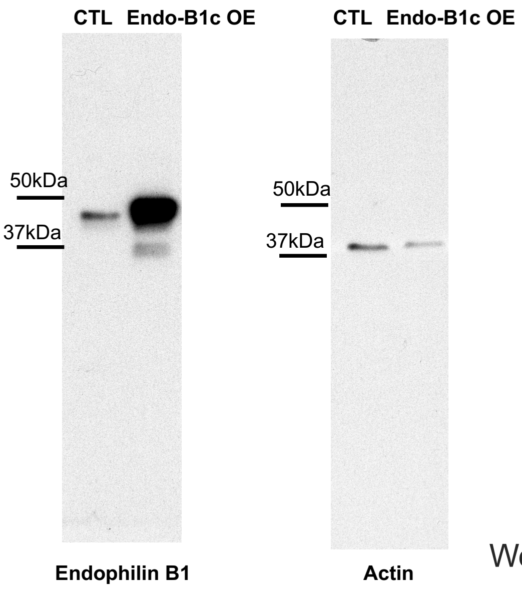

Western Blot: Fig 5

Supplemental Figure S2: Full Western blots for data shown in Figs. 1, 3, 4 and 5

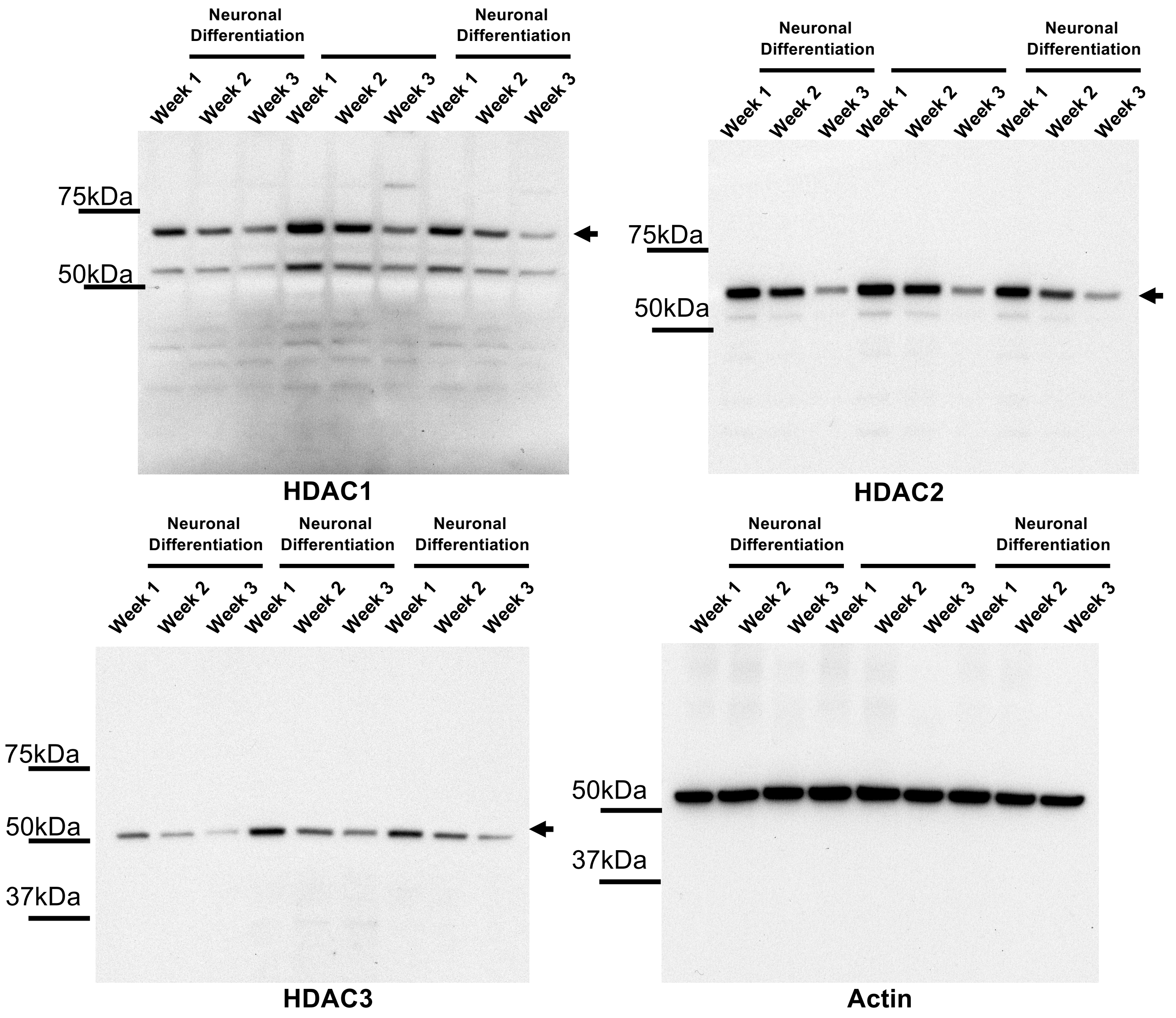

Supplemental Figure S3:
Full Western blots for data shown
in Supplemental Fig. S1
